# Changes of the *in vivo* kinematics of the human medial longitudinal foot arch, first metatarsophalangeal joint, and the length of plantar fascia in different running patterns

**DOI:** 10.3389/fbioe.2022.959807

**Published:** 2022-11-29

**Authors:** Xiaole Sun, Wanyan Su, Faning Zhang, Dongqiang Ye, Shaobai Wang, Shen Zhang, Weijie Fu

**Affiliations:** ^1^ School of Exercise and Health, Shanghai University of Sport, Shanghai, China; ^2^ School of Exercise and Health, Nanjing Sport Institute, Nanjing, China; ^3^ School of Athletic Performance, Shanghai University of Sport, Shanghai, China; ^4^ Key Laboratory of Exercise and Health Sciences of Ministry of Education, Shanghai University of Sport, Shanghai, China

**Keywords:** dual fluoroscopic imaging system, plantar fascia, medial longitudinal arch, first metatarsophalangeal joint, running

## Abstract

Accurately obtaining the *in vivo* motion of the medial longitudinal arch (MLA), first metatarsophalangeal joint (MTPJ), and plantar fascia (PF) is essential for analyzing the biomechanics of these structures in different running strike patterns. Most previous studies on the biomechanics of the MLA, first MTPJ, and PF have been based on traditional skin-marker–based motion capture, which cannot acquire the natural foot motion. Therefore, this study aimed to 1) describe the movement of the MLA, first MTPJ, and PF during running by using the high-speed dual fluoroscopic imaging system (DFIS) and 2) explore changes of the *in vivo* kinematics of the MLA and first MTPJ, and the length of the PF during the stance phase of running with different foot strike patterns. Fifteen healthy male runners all of whom ran with a regular rearfoot strike (RFS) pattern were required to run with forefoot strike (FFS) and RFS patterns. Computed tomography scans were taken from each participant’s right foot for the construction of 3D models (the calcaneus, first metatarsal, and first proximal phalanges) and local coordinate systems. A high-speed DFIS (100 Hz) and 3D force platform (2,000 Hz) were used to acquire X-ray images of the foot bones and ground reaction force data during the stance phase of running (3 m/s ± 5%) simultaneously. Then, 3D-2D registration was used to obtain the *in vivo* kinematic data of the MLA and first MTPJ and the length of the PF. When compared with RFS, in FFS, 1) the range of motion (ROM) of the medial/lateral (5.84 ± 5.61 mm *vs*. 0.75 ± 3.38 mm, *p* = 0.002), anterior/posterior (14.64 ± 4.33 mm *vs*. 11.18 ± 3.56 mm, *p* = 0.010), plantarflexion/dorsiflexion (7.13 ± 3.22° *vs*. 1.63 ± 3.29°, *p* < 0.001), and adduction/abduction (−3.89 ± 3.85° *vs*. −0.64 ± 4.39°, *p* = 0.034) motions of the MLA were increased significantly; 2) the ROM of the anterior/posterior (7.81 ± 2.84 mm *vs*. 6.24 ± 3.43 mm, *p* = 0.003), superior/inferior (2.11 ± 2.06 mm *vs*. −0.57 ± 1.65 mm, *p* = 0.001), and extension/flexion (−9.68 ± 9.16° *vs*. −5.72 ± 7.33°, *p* = 0.018) motions of the first MTPJ were increased significantly; 3) the maximum strain (0.093 ± 0.023 *vs*. 0.075 ± 0.020, *p* < 0.001) and the maximum power (4.36 ± 1.51 W/kg *vs*. 3.06 ± 1.39 W/kg, *p* < 0.001) of the PF were increased significantly. Running with FFS may increase deformation, energy storage, and release of the MLA and PF, as well as the push-off effect of the MTPJ. Meanwhile, the maximum extension angle of the first MTPJ and MLA deformation increased in FFS, which showed that the PF experienced more stretch and potentially indicated that FFS enhanced the PF mechanical responses.

## 1 Introduction

Running is an increasingly popular activity worldwide because it is one of the most accessible sports to achieve better physical health and disease prevention (Li et al., 2022). However, up to 79% of runners are afflicted by lower extremity injuries each year (Xu et al., 2021; Anderson et al., 2022). Plantar fasciitis is the third most common running injury accounting for approximately 10% in runners (Cosca and Franco, 2007; Kakouris et al., 2021). Understanding the injury mechanism of plantar fasciitis is the premise of how to reduce its injury incidence. Given the potential association between altered biomechanics during running and injury risk, it is plausible that changing running techniques can be either beneficial or detrimental in the prevention of running-related injuries (Anderson et al., 2022).

Some researchers proposed transitioning from rearfoot strike (RFS) to forefoot strike (FFS) during running to reduce the incidence of plantar fasciitis (Crowell and Davis, 2011; Williams et al., 2012). However, the biomechanical performance of the plantar fascia (PF) in the FFS and RFS conditions was different (McDonald et al., 2016; Chen et al., 2019a). Therefore, understanding the influence of foot strike patterns on the PF is the key to clarifying the link between foot strike patterns and the occurrence of injuries. Some studies have considered a potential association between the impact of vertical ground reaction force (vGRF) and plantar fasciitis, that greater external loads applied to the foot may subject the PF to abnormal mechanical loading, placing the structure at greater vulnerability to injury (Pohl et al., 2009; Johnson et al., 2020). In addition, insufficient foot muscle strength is also linked with plantar fasciitis (Warren and Jones, 1987; Latey et al., 2014; Cheung et al., 2016). As running with the FFS pattern has the potential to reduce the vGRF impact peak and strengthen foot muscles (Crowell and Davis, 2011; Williams et al., 2012; Shih et al., 2013), transitioning from RFS to FFS has received the most attention. However, some studies have shown that FFS may impose a bending strain to the medial longitudinal arch (MLA) and overstretch the PF, whereas RFS results in less MLA compression and PF stretch (Arangio et al., 1998; Lieberman et al., 2010; Chen et al., 2019a; Chen et al., 2019b). Conversely, McDonald et al. (2016) found that FFS would increase the activation of intrinsic foot muscles, which may reduce excessive compression of the MLA and PF strain. Therefore, investigating the effect of different foot strike running patterns on the length variation of the PF is a prerequisite for understanding the function of the PF during running.

The motion of the MLA and metatarsophalangeal joint (MTPJ) stretches the PF and changes its length due to their anatomical structure. With an origin at the calcaneal tuberosity and insertions at the base of each proximal phalanx, the PF is unique as it can be stretched *via* MLA compression and MTPJ extension (dorsiflexion) (Winter 1980; McDonald et al., 2016). Two mechanisms that explain these passive characteristics of the foot are the arch-spring and windlass mechanisms. The arch-spring proposed by Ker et al. (1987) conceptualizes the MLA as a dynamic truss with arch-spanning ligaments and the PF that serve as energy-saving springs (McDonald et al., 2016; Wager and Challis, 2016). In the windlass mechanism, proposed by Hicks (1954), the extension of MTPJ stiffens the PF whose tissue encapsulates small sesamoids inferior to the metatarsal heads. The sesamoids and PF slide around the metatarsal head, pulling the calcaneus toward the phalanges, shortening and raising the MLA (Hicks, 1954; Caravaggi et al., 2009). Consequently, the motion of the MLA and first MTPJ would change the length of the PF during running.

The quantification of the accurate movement of the MLA, MTPJ, and PF may contribute to further understand the influence of foot strike patterns on foot motion during running. At present, most studies explore the motion of the PF, MLA, and MTPJ by the traditional motion capture systems (Caravaggi et al., 2009; McDonald et al., 2016). Due to soft tissue artifacts, these systems cannot accurately track the 3D motion of the foot bones during locomotion. In addition, the PF is located deep in the skin of the foot, and some studies would use computational simulations such as OpenSim (McDonald et al., 2016; Wager and Challis, 2016) and finite element analysis (Chen et al., 2019b) to further obtain its motion, which is based on the position of skin markers placed on the calcaneus and head of the first metatarsal. Due to the simplification and assumption made in the modeling procedure and the single-subject design (Wager et al., 2016; Chen et al., 2019b), the computational simulations could amplify the measurement error. In recent years, the dual fluoroscopic imaging system (DFIS) has emerged as a viable tool to capture *in vivo* bone motion in sports analysis and medical rehabilitation. The system can noninvasively analyze individual joints with high reproducibility without being affected by the errors caused by the skin or soft tissues (Torry et al., 2011).

Therefore, the purpose of this study is to 1) describe the movement of the MLA, first MTPJ, and PF during running by using the high-speed DFIS and 2) explore the changes of *in vivo* kinematics of the MLA, first MTPJ, and length variation of the PF during running with different strike patterns. We hypothesized that 1) during the stance phase, the MLA would dorsiflex and then plantarflex with the PF strain increasing and then decreasing, while the extension angle of the first MTPJ would decrease and then increase, and 2) when compared with RFS, deformation of the MLA, the range of motion (ROM) of the first MTPJ extension/flexion, and PF strain were significantly increased in FFS conditions.

## 2 Materials and methods

### 2.1 Participants

Fifteen healthy male amateur runners (age: 32.7 ± 7.7 years, height: 172.9 ± 3.9 cm, weight: 72.9 ± 7.0 kg, and running distance: 39.1 ± 17.0 km/week) with an average of 4.7 ± 3.7 years of running experience were recruited to participate in this study. The sample size was determined through a G*power statistical calculation (with a statistical power of a = 0.05 and test power of 0.8) (Faul et al., 2007). The inclusion criteria were as follows: 1) habitual RFS runners, 2) right-foot dominant, defined as the preferred kicking leg (Niu et al., 2011), 3) running more than 20 km per week, 4) no lower limb injury in the past 6 months, and 5) no vigorous exercise within 24 h before the experiment. Before the test, the participants were familiarized with the experimental protocol and potential risks. An informed written consent was obtained from each participant and approved by the Institutional Review Board of Shanghai University of Sport (102772021RT034).

### 2.2 Experimental procedure

#### 2.2.1 Computed tomography scan

Each participant underwent CT scanning (SOMATOM, Siemens AG, Germany) of the right foot for the creation of the 3D bone models, which would be used in 3D-2D registration. During scanning, the participant lay supine with the plantar surface of their foot constrained at 90° from the scanning table (average resolution: 0.488 mm × 0.488 mm × 0.625 mm). The foot position was maintained during the scan *via* a custom-made foam support. The scanning scale ranged from 10 cm superior of the ankle joint to the bottom of the heel at 0.6 mm slice thickness, 318 mm field of view, 512 × 512 acquisition matrix, 120 kV, and 140 mA.

#### 2.2.2 Data collection

**FIGURE 1:**
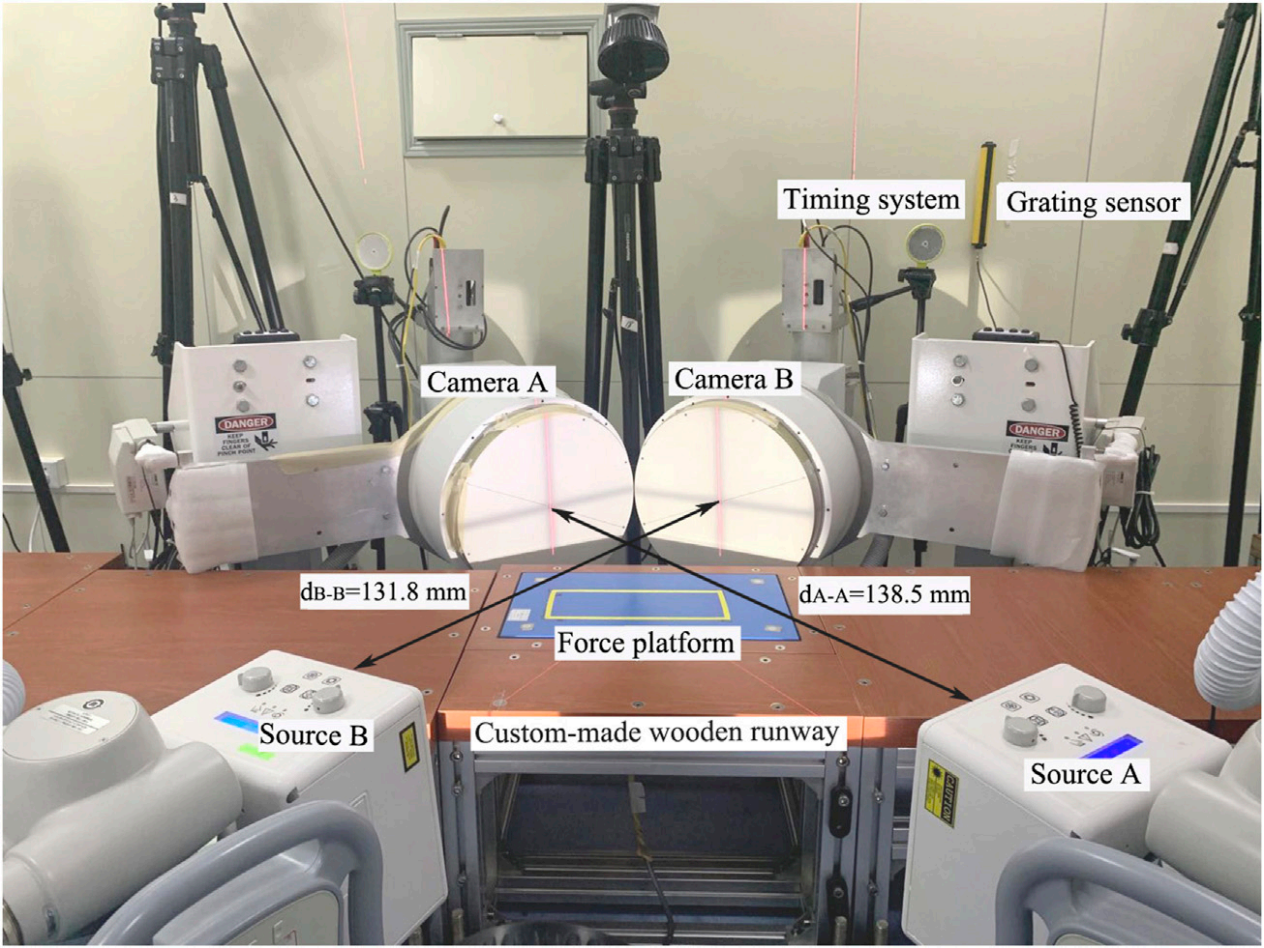
Experimental setup.

The high-speed DFIS has been previously validated by Zhang et al. (2022). The DFIS consists of two X-ray generators and two image intensifiers, optically coupled to synchronize high-speed video cameras. The system is configured with a 120° inter-beam angle and a source image distance of 131.8 and 138.5 cm for the two intensifiers (Figure 1), respectively. Radiographic images were acquired with the X-ray generators in a single radiographic mode (60 kVp, 63 mA, 1/1,000 s exposure speed). The image resolution for each X-ray image was 1,024 × 1,024. The X-ray images were collected for 0.6 s at 100 Hz when the subjects performed a running task, with the average dose rate of 0.08 mSv/100 frames (Zhang et al., 2021). For each participant, the maximum number of collected trials by the DFIS was 10 to reduce the radiation as much as possible, such that the maximum radiation dose for each participant from the DFIS was no more than 480 μSv. In addition, the estimated effective dose for foot and ankle CT (70 μSv) and the DFIS trials totaled 550 μSv (the maximum radiation), which is far below the annual occupational limit 50,000 μSv systemic effective dose limit set by the United States Nuclear Regulatory Commission (Cross et al., 2017).

Prior to the testing, the participants were required to warm up on a traditional treadmill at 3 m/s for 5 min and to familiarize with the target speed in the formal test. Subsequently, the participants were allowed as much time as required to become familiar with the raised custom-made wooden runway (710 cm × 60 cm × 47 cm, Figure 1) and with the selected starting position, which ensured that the participant’s right foot landed naturally in the middle of the X-ray volume. During the testing, each participant was required to run at 3 m/s (± 5%) with their preferred RFS along the runway. A RFS trial was completed successfully, then an FFS trial was collected. Meanwhile, the experimenter instructed the runners on how to run with FFS, requiring the runners to touch the ground with the ball of the foot initially and allowing the rearfoot to touch the ground subsequently (Lieberman et al., 2010; Roper et al., 2017). A rest interval of 20 s was allowed between the trials. During running, the bodies of the participants blocked the infrared blocking grating sensor and triggered the DFIS (100 Hz) and 3D force platform (9260AA3, Kistler Corporation, Switzerland, 2,000 Hz) to collect X-ray images of the foot bones and GRF data during the stance phase of running simultaneously. For each participant, one successful trial in which the foot was completely in the middle of the X-ray volume was included in each condition. During the test, all the participants wore the same experimental shoes (traditional running footwear: LI-NING LAN-ARHP171; heel-to-toe drop: 6 mm; midsole material: TPU, EVA; upper structure: textile fabric; and without any arch support). The strike pattern was classified as either FFS or RFS from the position where the foot touched the ground initially in the collected X-ray images (Welte et al., 2021) and whether there was an impact force peak of the curve of vGRF by the 3D force platform (Daoud et al., 2012).

### 2.3 Data processing

#### 2.3.1 Three-dimensional model

The calcaneus, first metatarsal, first proximal phalanx, and sesamoids were subsequently segmented from other bones and soft tissues (Mimics, Materialise, Leuven, Belgium) to provide 3D tessellated surface and partial volumes of the bones. The partial volumes were generated to create digitally reconstructed radiographs (Miranda et al., 2011). Smoothing and noise reduction were used to smooth the bone surface, the number of iterations was set to 2, and the smoothing factor was set to 0.4.

The two sesamoid bones and the first metatarsal were grouped, which were tracked as a single rigid body as it was difficult to track the *in vivo* kinematics of the sesamoids (Welte et al., 2021). Anatomic coordinate systems were created for the calcaneus, first metatarsal, and first proximal phalanx in the right foot using the inertial anatomical coordinate system, with the origin located at the centroid and the x–y–z axes aligned along the principal axis of the moment of the inertia tensor (Eberly et al., 1991). The axes were relabeled such that the x-axis was mediolateral, the y-axis was anteroposterior, and the z-axis was superioinferior (Welte et al., 2021).

**FIGURE 2:**
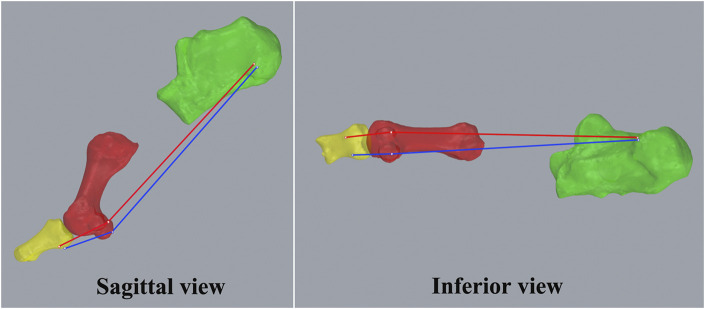
Models of the medial longitudinal arch (the first metatarsal—red and the calcaneus—green), first metatarsophalangeal joint (the first proximal phalanx—yellow, the first metatarsal), and plantar fascia (red and blue lines).

The MLA model was simplified to include the first metatarsal and calcaneus, while the first MTPJ model consisted of the first proximal phalanx and first metatarsal (Figure 2). The PF was modeled as two fibers connecting the origin, insertion, and sesamoid contact points of the first band of the PF. The origin was selected as two adjacent points on the medial one-fifth of the lateral tubercle of the calcaneus, and the insertion was selected on the medial and lateral insertion points of the phalanx for one participant (Bojsen-Moller and Flagstad, 1976; Sarrafian et al., 1983). The sesamoid contact points were located at the two most inferior points on the model of the medial and lateral sesamoid bones, respectively, in the z-axis direction of its anatomical coordinate system. The average length of the long line segments (between the origin and sesamoid contact point) and the short line segments (between the sesamoid contact point and insertion point) was the plantar fascia length (Figure 2) (Welte et al., 2021).

#### 2.3.2 Kinematic data

All radiographic images were undistorted using an “un-distortion” grid (Brainerd et al., 2010) and calibrated using a custom calibration cube in XMALab software (Brown University, United States) (Knörlein et al., 2016). The calibration object was used to determine the location of the two high-speed cameras and X-ray sources. The calibrated X-ray image depth and sharpening were performed (Adobe Photoshop 12.0, Adobe Systems Software Ireland Ltd, United States) to facilitate the identification of the outline of the bones during subsequent 3D-2D registration with an image depth of 16, an amount of 199%, a radius of 11.7 pixels, and a threshold of 0.

The position and orientation of each bone were determined *via* 3D-2D registration (Rhinoceros 6.0, McNeel & Associates, United States). The 3D-2D registration was a process by which the 3D partial volume of the bone of interest was virtually placed in the DFIS 3D environment. According to the outline of the bone in the image, the bone was translated and rotated manually in the 3D environment until the projected outline of the bone model matched the X-ray bone outline. This process yielded the *in vivo* kinematic data of the MLA and first MTPJ, and the length of the PF. The *in vivo* kinematic analysis of the MLA and first MTPJ were based on the motions of the first metatarsal coordinate system with respect to the calcaneus coordinate system in six directions, and the motions of the first proximal phalanx coordinate system with respect to the first metatarsal coordinate system in six directions, respectively. The medial (+)/lateral (M/L), anterior (+)/posterior (A/P), and superior (+)/inferior (S/I) directions were aligned with the x-, y-, and z-axes of the coordinate systems, respectively. The plantarflexion (+)/dorsiflexion (PF/DF), inversion (+)/eversion (IR/ER), and adduction (+)/abduction (AD/AB) were determined as rotations around the x-, y-, and z-axes of the coordinate systems, respectively (Wu et al., 2002). The *in vivo* kinematic data of the MLA and the length of the PF were normalized with the corresponding data of the MLA and PF for the neutral position of the foot (Gefen, 2003).

**FIGURE 3:**
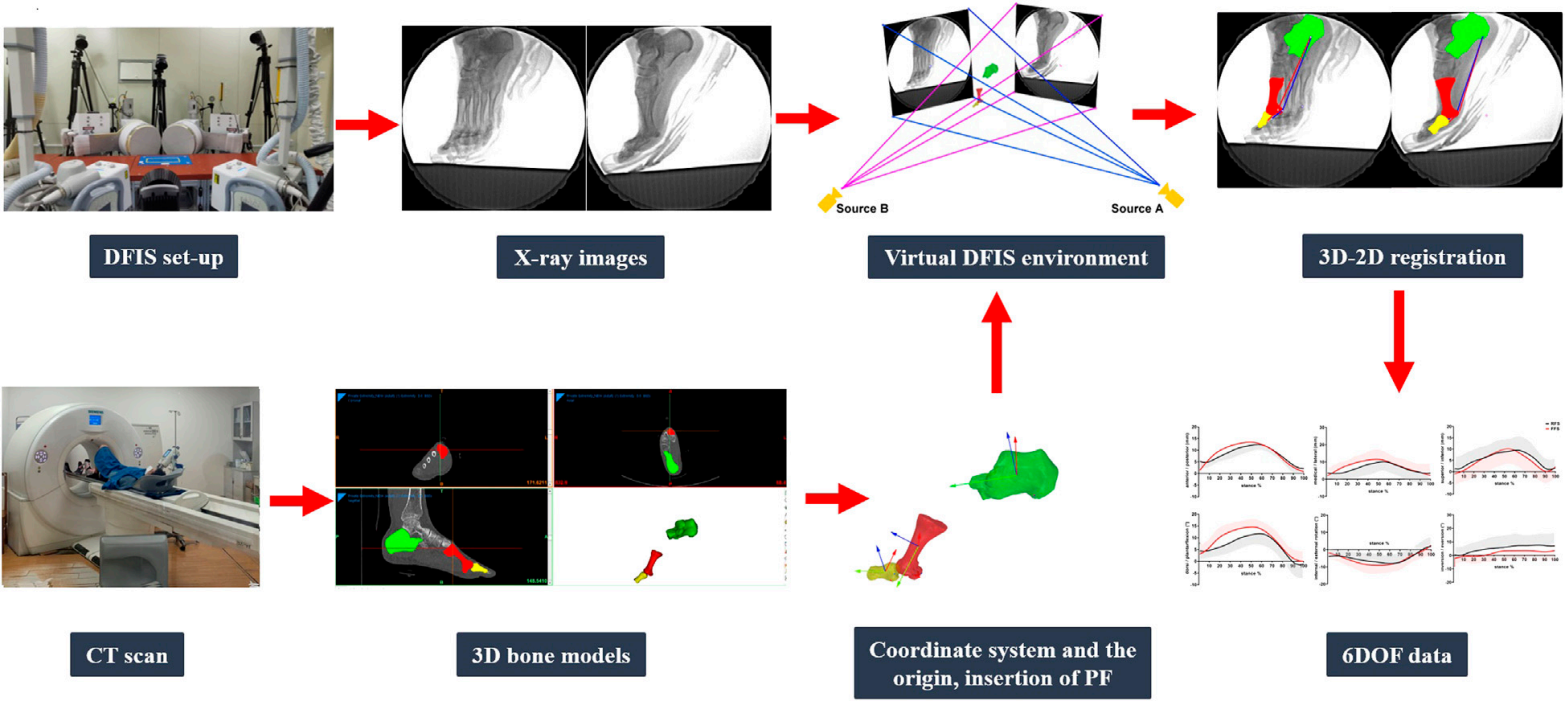
DFIS data collection and processing.

All data were time-normalized and filtered with an adaptive low-pass Butterworth filter with a cut-off frequency of 20 Hz (MATLAB, R2018a, MathWorks, Natick, United States). The stance phase was defined as the time interval from the instant of touchdown, which was determined as the instant vGRF exceeding a threshold of 15 N (Welte et al., 2018), to the instant of take-off (Yang et al., 2021). Specifically, the stance phase was divided into four phases: early stance (0–20%), mid-stance (20–55%), propulsion (55–85%), and late stance (85–100%) (Welte et al., 2021). Data collection and processing are shown in Figure 3.

### 2.4 Parameters

PF strain was defined as the difference between the PF instantaneous length (L_i_) and the length in the neutral position (L_neu_) divided by L_neu_. The PF power was defined as the product of PF force (F_PF_) and velocity (V_PF_). Force data were obtained using a stiffness value of 187 N/mm multiplied by the length change of the PF (ΔL_i_). Velocity was defined as the change in PF length with respect to the change in time. PF strain and power were calculated as follows:

PF strain = ( $L_{i}-L_{neu})/L_{neu}$ ×100% (Gefen, 2003), PF power = F_PF_ ×V_PF_ =( 187N/mm × ΔL_i_)×V_PF_ (McDonald et al., 2016).

The *in vivo* kinematics of the MLA and first MTPJ included the maximum and minimum data in six directions (M/L, A/P, S/I, PF/DF, IR/ER, and AB/AD); the ROM of the MLA and first MTPJ was defined as the differences between the maximum and minimum data in six directions; PF length change was defined as the difference between the maximum and minimum length during the four phases and the entire stance phase; and the maximum PF strain.

### 2.5 Statistics

The distribution of all dependent variables conforming to a normal distribution was examined by the Shapiro–Wilk test. The distribution did not differ significantly from normality. The mean and standard deviation for each variable were calculated. The changes of the *in vivo* kinematic data of the MLA and first MTPJ and the length of the PF during running with foot strike patterns were determined through a paired sample t-test (SPSS 25.0, IBM, Chicago, United States). The significance level α was set at 0.05.

## 3 Results

### 3.1 Global motion characteristics

**FIGURE 4:**
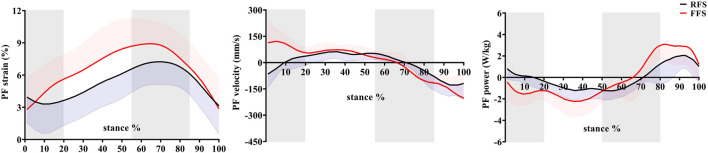
Effects of foot strike patterns on strain, velocity, and power of the plantar fascia during the stance phase of running. Note: RFS, rearfoot strike pattern and FFS, forefoot strike pattern.

**FIGURE 5:**
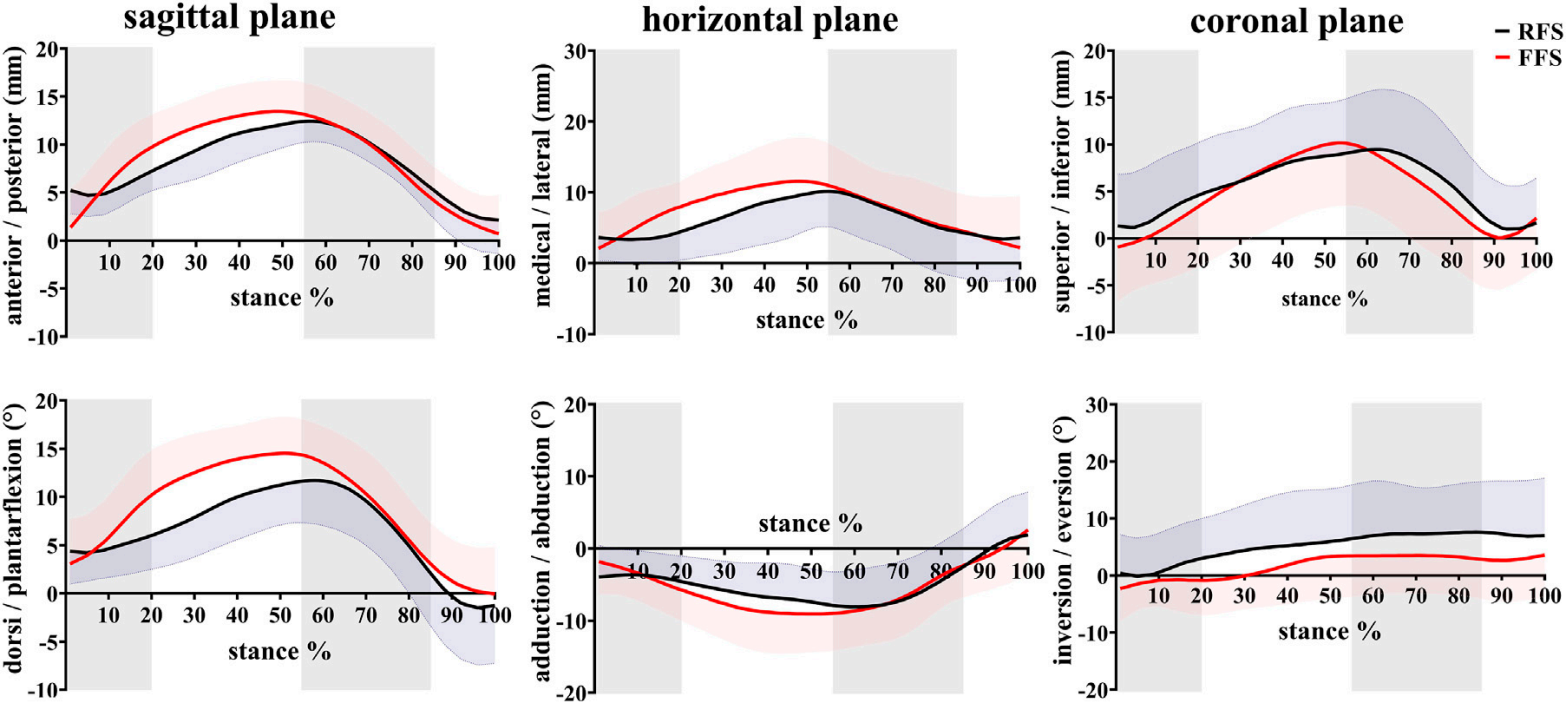
Effects of different foot strike patterns on the *in vivo* kinematics data of MLA during the stance phase of running. Note: RFS, rearfoot strike pattern and FFS, forefoot strike pattern.

**FIGURE 6:**
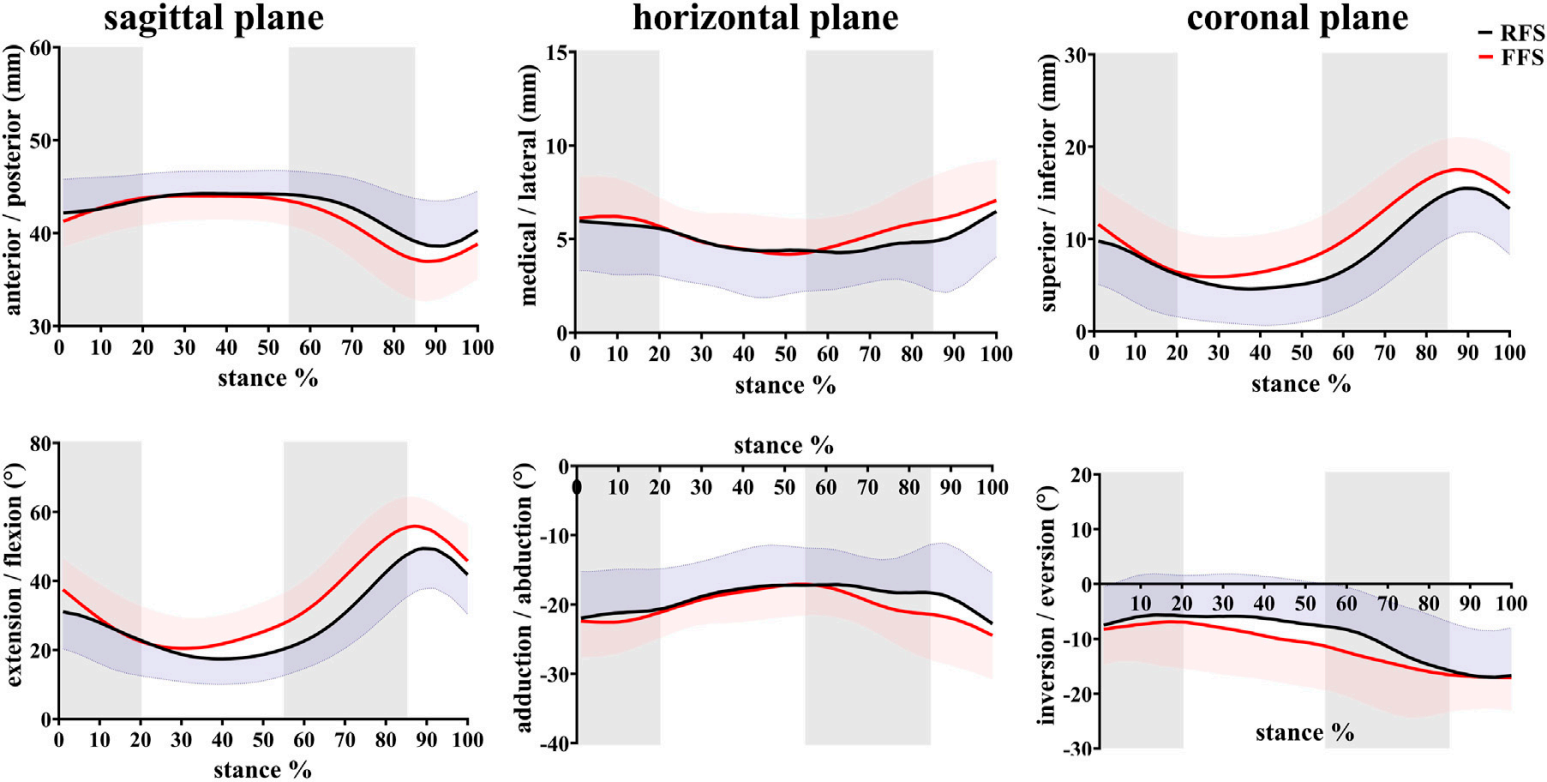
Effects of different foot strike patterns on the *in vivo* kinematics data of the first MTPJ during the stance phase of running. Note: RFS, rearfoot strike pattern and FFS, forefoot strike pattern.

During the early stance (0–20%), the first MTPJ primarily flexed, during which the PF generally lengthened slowly (FFS) or remained quasi-isometric (RFS), and the MLA dorsiflexed. During mid-stance (20–55%), the first MTPJ angle changed minimally when the toes were flat, during which the PF elongated rapidly, and the MLA continued to dorsiflex and abduct. During the propulsion phase (55–85%), the first MTPJ extended, with MLA plantarflexion and adduction, and the PF lengthened and then shortened. During the late stance phase (85–100%), the first MTPJ flexed, with MLA plantarflexion, and the PF shortened (Figures 4–6).

### 3.2 Angles and range of motion

**FIGURE 7:**
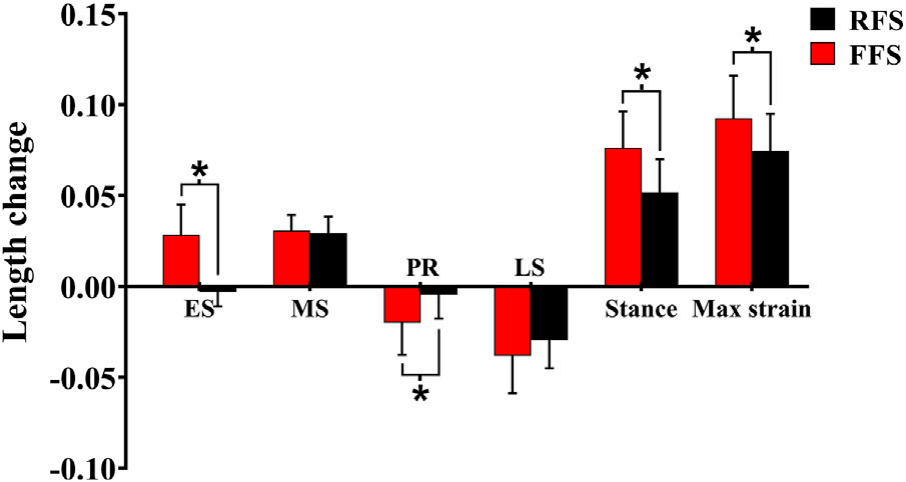
Effects of different foot strike patterns on the length change, peak strain of the plantar fascia during the stance phase of running. Note: RFS, rearfoot strike pattern and FFS, forefoot strike pattern; ES, early stance phase; MS, mid-stance phase; PR, propulsion phase; LS, late stance phase; stance, entire stance phase; max strain, the maximum strain of the plantar fascia. * significant difference between FFS and RFS during running, *p* < 0.05.

When compared with RFS, the changes in the length of the PF during the early stance phase (*p* < 0.001), the propulsion phase (*p* = 0.001), and the entire stance phase (*p* < 0.001) were significantly increased in the FFS condition. The maximum strain (*p* < 0.001) of the PF was also significantly increased in FFS (Figure 7). The maximum power of the PF (*p* < 0.001) was greater in the FFS condition (Figure 4).

**FIGURE 8:**
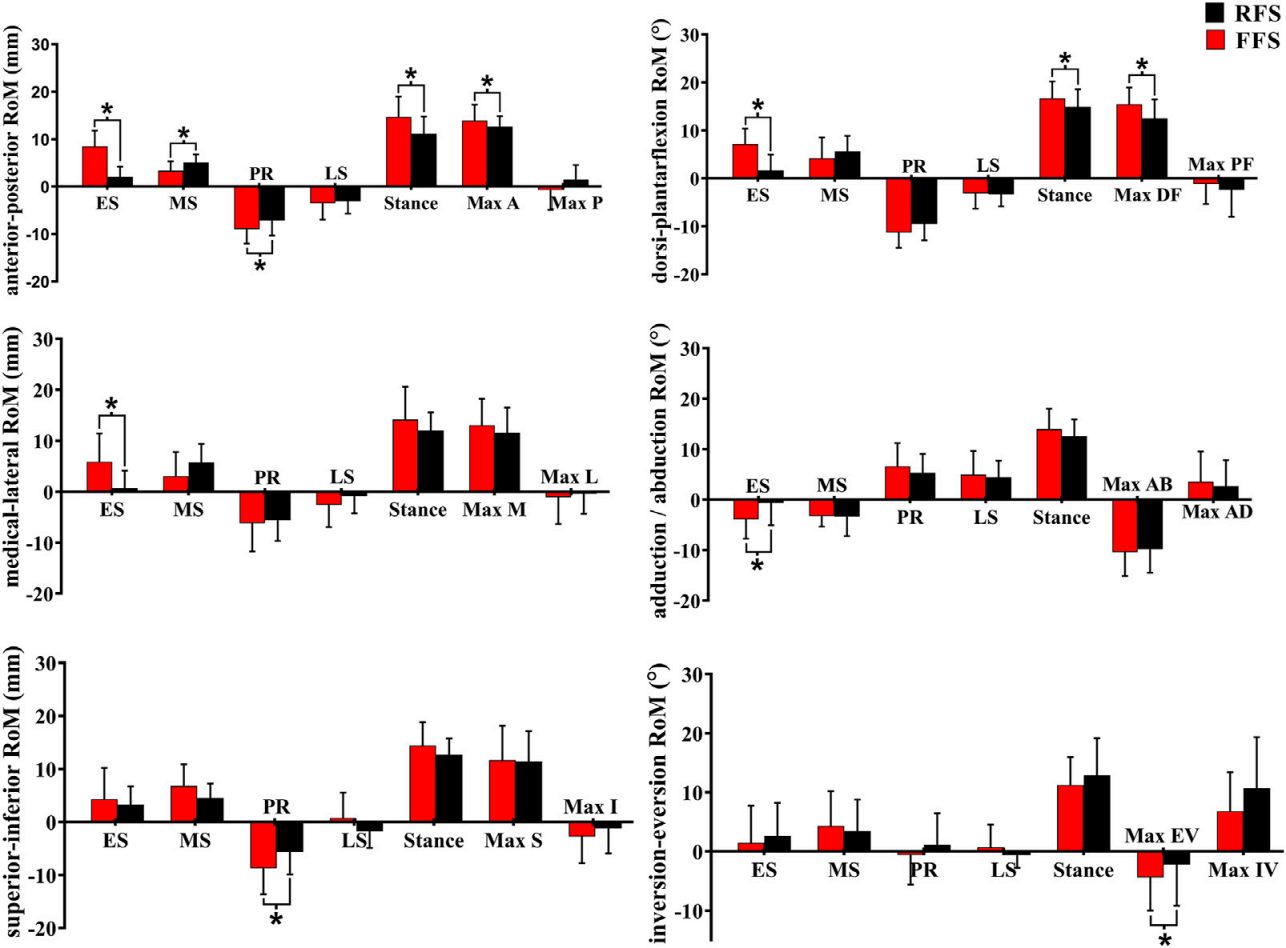
Effects of different foot strike patterns on the range of motion of MLA and characteristic values during the stance phase of running. Note: RFS, rearfoot strike pattern and FFS, forefoot strike pattern; ES, early stance phase; MS, mid-stance phase; PR, propulsion phase; LS, late stance phase; stance, entire stance phase; A, anterior translation; P, posterior translation; DF, dorsiflexion; PF, plantarflexion; M, medial translation; L, lateral translation; AD, adduction; AB, abduction; S, superior translation; I, inferior translation; EV, eversion; IV, inversion. * significant difference between FFS and RFS during running, *p* < 0.05.

In the translation of the MLA, when compared with RFS, the medial/lateral translation ROM during the early stance phase was significantly increased in FFS (*p* = 0.002). The anterior/posterior translation ROM during the early stance phase (*p* < 0.001), the propulsion phase (*p* = 0.012), and the entire stance phase (*p* = 0.010) was significantly increased in FFS. The peak anterior translation during the entire stance phase (*p* = 0.028) and the superior/inferior translation during the propulsion phase (*p* = 0.002) was significantly increased in FFS. In the rotation of the MLA, when compared with RFS, the plantarflexion/dorsiflexion ROM (*p* < 0.001), peak dorsiflexion angle (*p* = 0.017), adduction/abduction ROM (*p* = 0.034), and magnitude of the maximum MLA compression (*p* = 0.006) were significantly increased in FFS. However, the peak inversion angle was significantly smaller (*p* = 0.042) in FFS (Figure 8).

**FIGURE 9:**
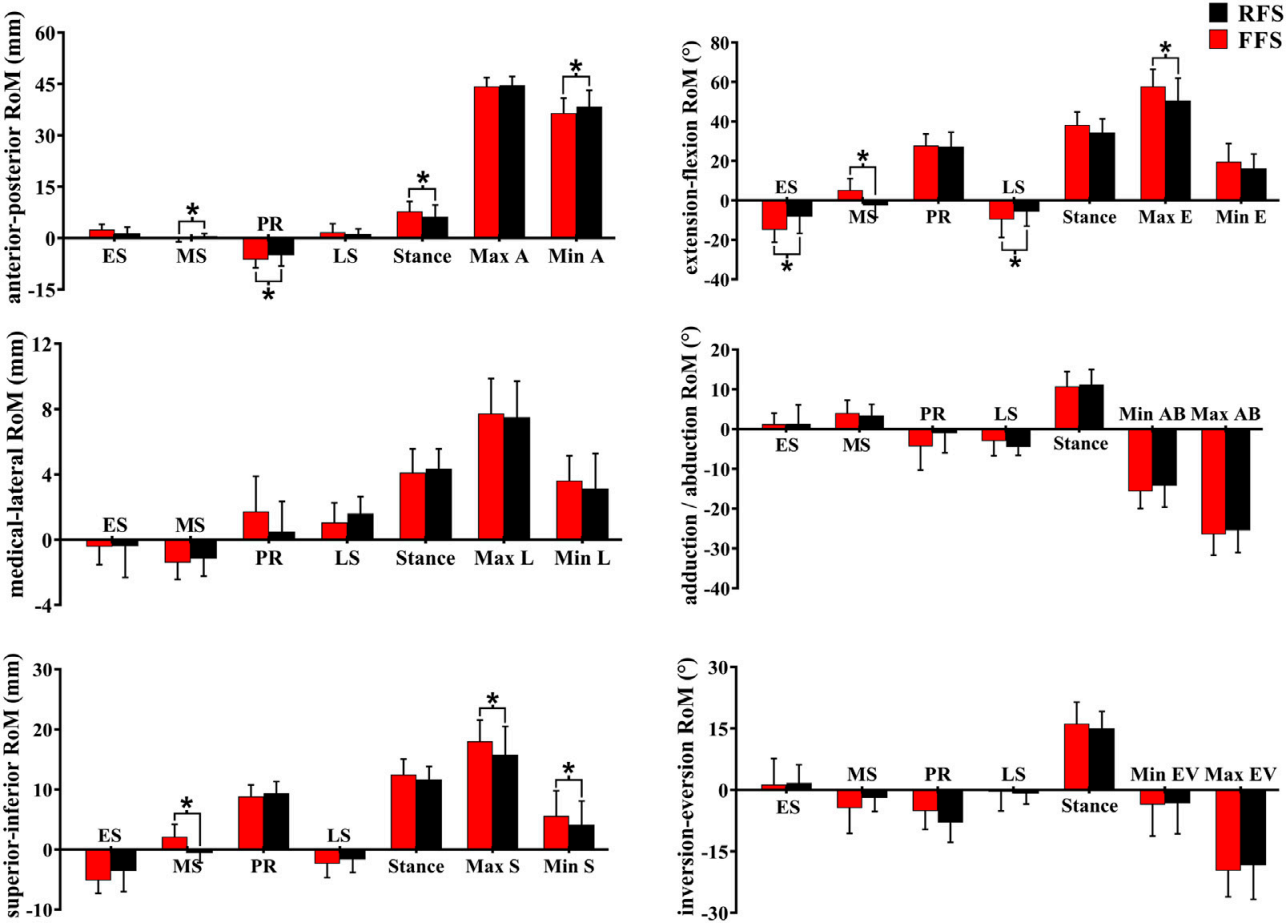
Effects of different foot strike patterns on the ROM of the first MTPJ and characteristic values during the stance phase of running. Note: RFS, rearfoot strike pattern and FFS, forefoot strike pattern; ES, early stance phase; MS, mid-stance phase; PR, propulsion phase; LS, late stance phase; stance, entire stance phase; A, anterior translation; P, posterior translation; E, extension; M, medial translation; L, lateral translation; AD, adduction; AB, abduction; S, superior translation; I, inferior translation; EV, eversion; IV, inversion. * significant difference between FFS and RFS during running, *p* < 0.05.

In the translation of the first MTPJ, when compared with RFS, the anterior/posterior ROM during the propulsion phase (*p* = 0.008) and the entire stance phase (*p* = 0.003), the superior/inferior ROM during the mid-stance phase (*p* = 0.001), maximum superior translation (*p* = 0.002), and maximum inferior translation (*p* = 0.04) were significantly increased in FFS. In rotation motion, when compared with RFS, the flexion/extension ROM during the early stance phase (*p* = 0.006), mid-stance phase (*p* = 0.002), and propulsion phase (*p* = 0.018) was significantly increased in FFS. The maximum extension angle (*p* < 0.001) and the extension angle at initial contact (*p* = 0.004) were significantly larger in FFS (Figure 9).

## 4 Discussion

This study investigated the changes of the *in vivo* kinematics of the MLA and first MTPJ and the length of the PF during the stance phase of running with FFS and RFS on the basis of the DFIS. The results showed that when compared with RFS, the ROM of the MLA (medial/lateral, anterior/posterior, and adduction/abduction) and first MTPJ (anterior/posterior, superior/inferior, and plantarflexion/dorsiflexion) and the length of the PF (maximum strain, and power) were significantly increased in FFS. These results indicate that running with the FFS pattern may enhance PF mechanical responses and deformation of the MLA potentially. These results are consistent with our hypothesis.

This study found that in FFS and RFS, PF strain gradually increased from the initial contact and reached a maximum of ∼9% at ∼65% of the stance phase, during which the PF stored elastic energy (negative power). Then, PF strain gradually decreased, during which the PF released elastic energy (positive power). This finding shows that the PF conforms to the general stretch–shorten model of the elastic structure. Similarly, Wager et al. (2016) found that regardless of RFS or non-RFS, PF strain gradually increased until it reached the maximum of about 6% during the propulsion phase and then decreased. Meanwhile, we found that the motion trends were generally similar among the sagittal plane motion of the MLA and first MTPJ, and PF strain, which is consistent with the McDonald et al.’s study (2016). The study suggested that the windlass mechanism is generally present during running, wherein the increased dorsiflexion angle of the first MTPJ tightens the PF such that the PF acts like a windlass during the propulsion phase (Cheng et al., 2008a). In addition, during the early propulsion phase (55–70% stance), the PF length was quasi-isometric, that is, its strain was almost constant, while the first MTPJ experienced substantial dorsiflexion, and the MLA rose. The phase is known as the “forward windlass” (Welte et al., 2021). At this stage, the PF plays a role in resisting the tendency of the mid-foot to break in response to the body’s weight, such that the foot becomes a stable base of support. If the mid-foot was broken after heel rise, the MLA length or dorsiflexion angle would increase. However, we observed that the MLA was raised, and the anterior/posterior translation and dorsiflexion angle decreased. These findings have indicated that the PF and forward windlass contribute to stabilizing the foot by preventing mid-foot break. During the loading phase, the PF not only maintains the MLA morphology but also absorbs the impact energy through its deformation (Ker et al., 1987; Snow et al., 1995).

For the effects of foot strike patterns on the PF, we found that when compared with RFS, the strain (10–80% of the stance phase) and maximum strain of the PF were significantly increased in FFS, and the magnitude of PF length change during the early stance, propulsion phase, and entire stance phases was significantly enhanced in FFS. The results of this study are consistent with those of previous studies. Perl et al. (2012) found that when compared with RFS, PF strain and MLA deformation were significantly increased in FFS. However, McDonald et al. (2016) and Wager et al. (2016) found no significant difference in maximum strain of PF with different strike patterns. The difference might be due to the use of the motion capture system and further analysis of the effects of the foot strike patterns with the barefoot condition by OpenSim modeling. The peak strain of the PF primarily determines the resulting elastic energy benefits. This study found greater peak strain during FFS, which suggested that runners would receive additional performance benefits from the PF during FFS, which might improve their performance. Combined with the data on PF power, this study also found that PF power was greater during the early, mid-, and late stance phases in FFS, indicating that the PF absorbed and released more energy in FFS. Our finding of increased PF strain during FFS supported earlier findings which suggested that unaccustomed FFS may place more demands on the PF. Therefore, strengthening their foot muscles was required to reduce the risk of plantar fasciitis (Kogler et al., 2001; McKeon et al., 2015). In addition, we found that the PF shortened in RFS at the first 10% of the stance phase, but sustained elongation during this period in FFS. The preloading of the PF in FFS would change its tension and MLA length (Iwanuma et al., 2011), which also contributed to the forward movement of the body during the propulsion phase (Pataky et al., 2008). As this preloading reduced the curling of the PF collagen tissue, the strengthening of MLA stiffness occurred earlier, which helped transmit a greater push-off force to the ground during the propulsion phase (Caravaggi et al., 2009).

This study found that when compared with RFS, the ROM of the MLA in the sagittal plane (anterior/posterior and plantarflexion/dorsiflexion) was increased in FFS. These results have indicated that the compression of the MLA in the sagittal plane was significantly increased in FFS, which could store more elastic energy and attenuate impact. Similarly, the results of Perl et al. (2012) and Ardigò et al. (1995) are consistent with our findings. It is possibly the vGRF located anterior to the center of the ankle at the initial contact in FFS which generated a high Achilles tendon tension (Landreneau et al., 2014; Wong et al., 2014). Combined with the body weight acting on the ankle, the MLA initially loaded in three-point bending from the instant the ball of the foot contacted the ground (Lieberman et al., 2010; Chen et al., 2019b). In RFS, during the loading phase, the GRF is located on the posterior of the MLA and inferior of the ankle, and the force of the tibialis anterior muscle acts on the medial cuneiform bone at the highest point of the MLA. As a result, there is less deformation in the MLA. Meanwhile, these forces improve the stiffness of the MLA before the mid-stance phase and prevent the MLA from absorbing the energy generated by the impact (Perl et al., 2012). Conversely, Wager et al. (2016) found no significant effect of foot strike patterns on MLA deformation during running. The inconsistency with the results of this study may have been caused by gender differences (males *vs*. females + males) and sample size (15 *vs*. 8). Normally, females demonstrate less arch height index and a greater arch motion than males, which could contribute to arch kinematics variation in genders (Takabayashi et al., 2020; Fukanoet al., 2012). In addition, the effects of foot strike patterns on arch kinematics may not be eliminated by the sample of three rearfoot and five forefoot strikers in Wager et al.’s. study. For the movement of the MLA in the horizontal plane, the ROM of the medial/lateral and adduction/abduction during the early stance phase was significantly greater in FFS. The increase of the MLA ROM in the horizontal plane during the stance phase may increase the energy loss.

As the distal part of the foot, the extension of the MTPJ was accomplished by the contraction of the toe flexor and plantar flexor muscles (Zhang et al., 2022). In this study, the first MTPJ was at an extended state during the entire stance phase of running with RFS and FFS, which is consistent with a previous study (Welte et al., 2021). It has been proven that the joint energy was absorbed in the first MTPJ and almost no energy was generated (Roy and Stefanyshyn, 2006). In RFS, the flexion/extension and superior/inferior ROM during the early stance, mid-stance, and propulsion phase were significantly decreased. The smaller joint ROM might result in less energy loss in the first MTPJ (Roy and Stefanyshyn, 2006). However, no significant differences were found in the ROM of the first MTPJ during the entire stance phase between the RFS and FFS conditions, which indicates that the effects of the strike patterns on energy absorption should be investigated in the future study. This study found that in the FFS condition, the ROM of the anterior/posterior and flexion/extension, maximum superior, and inferior translation during the propulsion phase was significantly increased, which indicated that the ROM of the first MTPJ was greater. By finite element analysis (Cheng et al., 2008b) and the cadaver experiment (Flanigan et al., 2007), previous studies found that the extension motion of the MTPJ could influence the tension and length of the PF; specifically, the larger extension angle would induce increased PF tension and length. Thus, the increased extension ROM and maximum extension angle of the first MTPJ in FFS indicated that the PF experienced more stretch. However, whether it was beneficial or detrimental in the prevention of plantar fasciitis needs to be further investigated. Due to the windlass mechanism, the larger extension of the first MTPJ could increase the stiffness of the MLA and enhance the push-off effect.

However, there were still several limitations in this study. First, only the kinematic parameters were analyzed. The kinetic data were not further explored. Second, the PF model was simplified as two-line segments, in which the distal part of the line segment would cross the sesamoid bone. The PF started at the calcaneus tuberosity wrapped around the sesamoid bone and ended at the proximal phalanx, without crossing the sesamoid bone. Third, the habitual RFS runners were unaccustomed to running with FFS. Even though practice time was given, habituation of the foot-strike technique may affect the kinematics characteristics. Fourth, one trial per condition per participant might not have been representative. Therefore, that might increase the discreteness of the *in vivo* kinematic data and reduce the accuracy of the results. Finally, the participants were healthy male runners. Only male participants were recruited because we failed to recruit enough women runners who met the inclusion criteria. However, we believe that the results of our study are still general enough and will inform the field. The further studies about the effects of different sexes, ages, and plantar fasciitis on the running performance need to be investigated.

## 5 Conclusion

This study found that when compared with RFS, the motion of the MLA in anterior/posterior translation and plantarflexion/dorsiflexion, and the ROM of the first MTPJ in anterior/posterior, superior/inferior translation, and extension/flexion during the stance phases of running were significantly increased in the FFS condition. These findings indicate greater deformation, more stored and released energy of the MLA, and better push-off effect of the MTPJ in the FFS condition. Meanwhile, maximum strain and the power of the PF were also increased significantly in the FFS condition, which indicated that the PF experienced more stretch and would store and release more elastic energy. When considering the PF mechanical responses, the kinematic properties of the MLA and MTPJ should be fully considered.

## Data Availability

The raw data supporting the conclusions of this article will be made available by the authors, without undue reservation.
